# 3-Ferrocenyl-2-(4-nitro­phen­yl)acrylonitrile

**DOI:** 10.1107/S160053681103306X

**Published:** 2011-08-27

**Authors:** Vincent O. Nyamori, Narainamah N. Moodley

**Affiliations:** a School of Chemistry, University of KwaZulu-Natal, Westville Campus, Private Bag X54001, Durban, 4000, South Africa

## Abstract

In the title compound, [Fe(C_5_H_5_)(C_14_H_9_N_2_O_2_)], the ferrocenyl rings exhibit an eclipsed conformation with a staggering angle of 15.9°, which is quite large compared to similar compounds.

## Related literature

For background to ferrocene chemistry, see: Štěpnička (2008 ▶); Gooding *et al.* (1983 ▶); Togni & Hayashi (1995 ▶). For related ferrocenylacrylonitrile structures, see: Cao & Ye (2008 ▶); Imrie *et al.* (2007 ▶). For the synthesis of acrylonitriles, see: Liu *et al.* (2001 ▶); Jeffery (1999 ▶); El-Tammany *et al.* (1983 ▶); Imrie *et al.* (2007 ▶).
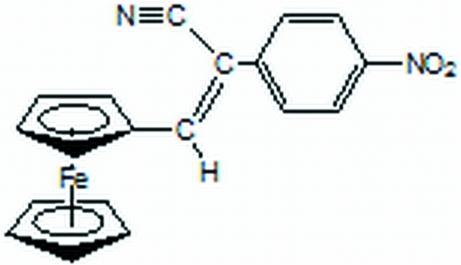


## Experimental

### 

#### Crystal data

[Fe(C_5_H_5_)(C_14_H_9_N_2_O_2_)]
*M* *_r_* = 358.17
Monoclinic, 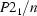

*a* = 6.7186 (14) Å
*b* = 28.036 (6) Å
*c* = 8.4165 (18) Å
β = 90.108 (6)°
*V* = 1585.3 (6) Å^3^
*Z* = 4
Mo *K*α radiation
μ = 0.96 mm^−1^
*T* = 173 K
0.53 × 0.05 × 0.02 mm

#### Data collection

Bruker APEXII CCD diffractometer
Absorption correction: multi-scan (*SADABS*; Bruker, 2005 ▶) *T*_min_ = 0.629, *T*_max_ = 0.981
9108 measured reflections
2672 independent reflections
1437 reflections with *I* > 2σ(*I*)
*R*_int_ = 0.112

#### Refinement

*R*[*F* ^2^ > 2σ(*F* ^2^)] = 0.051
*wR*(*F* ^2^) = 0.114
*S* = 0.90
2672 reflections
218 parameters
H-atom parameters constrained
Δρ_max_ = 0.36 e Å^−3^
Δρ_min_ = −0.65 e Å^−3^

### 

Data collection: *APEX2* (Bruker, 2005 ▶); cell refinement: *SAINT-Plus* (Bruker, 2005 ▶); data reduction: *SAINT-Plus*; program(s) used to solve structure: *SHELXS97* (Sheldrick, 2008 ▶); program(s) used to refine structure: *SHELXL97* (Sheldrick, 2008 ▶); molecular graphics: *SHELXTL* (Sheldrick, 2008 ▶); software used to prepare material for publication: *SHELXTL*.

## Supplementary Material

Crystal structure: contains datablock(s) global, I. DOI: 10.1107/S160053681103306X/gw2107sup1.cif

Structure factors: contains datablock(s) I. DOI: 10.1107/S160053681103306X/gw2107Isup2.hkl

Additional supplementary materials:  crystallographic information; 3D view; checkCIF report

## Figures and Tables

**Fig. 1.**
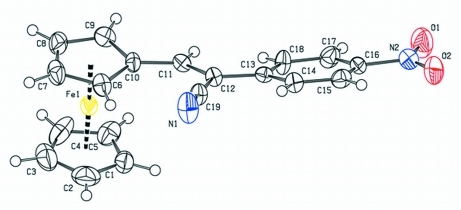
View of (I) (50% probability displacement ellipsoids).

## supplementary crystallographic information

### Comment

The synthesis of acrylonitrile compounds has previously been achieved by the use of Wittig reactions (Liu *et al.*, 2001), Heck reactions (Jeffery *et al.*, 1999) and McMurry coupling reactions (El-Tammany *et al.*, 1983). The growing interest in their synthesis and of their related derivatives, especially those with ferrocenyl moiety incorporated, is due to the fact that they are known to be important substrates for applications in material science (Štěpnička, 2008 and Togni & Hayashi, 1995). One of the major driving forces into their research is due to their ability to generate new materials with large second-order non-linear optical (NLO) features which in turn makes them useful in the field such application as photoactive semiconductors (Gooding, *et al.* 1983). The solvent-free reaction involved adding equimolar quantities of ferrocenecarboxaldehyde and 4-nitrophenylacetonitrile into a Pyrex tube. A catalytic amount of piperidine was added to act as a base. The reagents were thoroughly mixed, ground and then allowed to dry in the open air before being analysed by use of IR spectroscopy, 1*H*- and 13 C-NMR spectroscopy to determine the reaction progress. This reaction occurred readily and was characterized by the formation of a dark purple melt. The favourable reaction is due to the strong inductive effect of the nitro group which contributes to the activation of the methylene group taking part in the reaction. This could be explained by the kind of mechanism that operates in the Knoevenagel reaction where the strong electron withdrawing group (NO2 and CN) is expected to stabilize the intermediate. The molecule is characterized by two planes *i.e.* nitrile-ethylene and nitro-phenyl moieties which are almost coplanar with the torsion angles of C19–C12–C13–C14 being -8.8 (7) while C19–C12–C13–C18 is 6.0 (7)°. The nitro group of the nitrophenyl moiety is slightly twisted from the plane of the phenyl ring, with the torsion angles of C17–C16–N2–O1 and C15–C16–N2–O2 being 6.9 (7) and 6.5 (7) respectively. The ferrocenyl rings have an eclipsed conformation with a staggering angle of 15.9° which is quite large compared to a similar compound such as 3-ferrocenyl-2-(4-cyanophenyl)acrylonitrile (Imrie *et al.*, 2007) with an angle of 1.9°. The single C–C bonds around the ethylene group have a bond distance of 1.427 (7) Å for the C10–C11, which is shorter than C12–C14, 1.475 (7) Å. This difference in bond lengths has also been observed by Cao *et al.* (2008) and Imrie *et al.* (2007).

### Experimental

Into a Pyrex tube fitted with a ground glass joint, was added an equimolar quantity of ferrocenecarboxaldehyde (200.2 mg, 0.9353 mmol) and 4-nitrophenylacetonitrile (150.7 mg, 0.9294 mmol). The compounds were thoroughly mixed and ground. Thereafter, two drops of piperidine was added to the solid mixture. The reaction mixture rapidly changed into a purple melt which was allowed to dry into a dark purple solid as a crude product. The product was further subjected to column chromatography using hexane/diethyl ether (4:1) as the eluting solvent to achieve purple crystals (330 mg, 99%) m.p. 195°C; IR (KBr cm-1) 3096, 3056, 2218, 1602, 1579, 1511, 1456, 1371, 1253, 1199, 1199, 1049, 1032, 1001, 847, 823, 752, 689; 1*H*-NMR (CDCl3) 8.29 (2*H*, d, J 8.9, ArH), 7.77 (2*H*, d, J 8.9, ArH), 7.61 (1*H*, s, CH), 5.05 (2*H*, t, J 1.8, C5H4), 4.68 (2*H*, t, J 1.8, C5H4), 4.29 (5*H*, s, C5H5); 13 C-NMR (CDCl3) 147.87, 147.31, 141.35, 125.89, 124.87, 118.77, 104.41, 76.90, 73.21, 71.21, 70.59; m/*z* (EI) 359 (*M*+ +1, 12%), 358 (*M*+, 55%), 312 (7), 191 (6), 190 (12), 165 (6), 121 (25), 56 (6), 32 (19), 28 (100); Anal. Calc for C19H14N2O2Fe: C, 63.7; H, 3.9; N, 7.8; [*M*+], 358.040467; Found: C, 64.2; H, 4.3; N, 7.5; [*M*+], 358.040422.

### Refinement

The aromatic H atoms were placed in geometrically idealized positions and constrained to ride on their parent atoms, with C—H = 0.95 Å and *U*_iso_(H) = 1.2*U*_eq_(C).

### Crystal data

**Table:**

[Fe(C_5_H_5_)(C_14_H_9_N_2_O_2_)]	*F*(000) = 736
*M**_r_* = 358.17	*D*_x_ = 1.501 Mg m^−^^3^
Monoclinic, *P*2_1_/*n*	Mo *K*α radiation, λ = 0.71073 Å
Hall symbol: -P 2yn	Cell parameters from 1007 reflections
*a* = 6.7186 (14) Å	θ = 2.5–22.0°
*b* = 28.036 (6) Å	µ = 0.96 mm^−^^1^
*c* = 8.4165 (18) Å	*T* = 173 K
β = 90.108 (6)°	Needle, brown
*V* = 1585.3 (6) Å^3^	0.53 × 0.05 × 0.02 mm
*Z* = 4	

### Data collection

**Table:**

Bruker APEXII CCD diffractometer	1437 reflections with *I* > 2σ(*I*)
graphite	*R*_int_ = 0.112
φ and ω scans	θ_max_ = 25°, θ_min_ = 0.7°
Absorption correction: multi-scan (*SADABS*; Bruker, 2005)	*h* = −7→7
*T*_min_ = 0.629, *T*_max_ = 0.981	*k* = −24→33
9108 measured reflections	*l* = −10→9
2672 independent reflections	

### Refinement

**Table:**

Refinement on *F*^2^	Primary atom site location: structure-invariant direct methods
Least-squares matrix: full	Secondary atom site location: difference Fourier map
*R*[*F*^2^ > 2σ(*F*^2^)] = 0.051	Hydrogen site location: inferred from neighbouring sites
*wR*(*F*^2^) = 0.114	H-atom parameters constrained
*S* = 0.90	*w* = 1/[σ^2^(*F*_o_^2^) + (0.041*P*)^2^] where *P* = (*F*_o_^2^ + 2*F*_c_^2^)/3
2672 reflections	(Δ/σ)_max_ = 0.001
218 parameters	Δρ_max_ = 0.36 e Å^−^^3^
0 restraints	Δρ_min_ = −0.65 e Å^−^^3^

### Special details

**Table:**

Geometry. All e.s.d.'s (except the e.s.d. in the dihedral angle between two l.s. planes) are estimated using the full covariance matrix. The cell e.s.d.'s are taken into account individually in the estimation of e.s.d.'s in distances, angles and torsion angles; correlations between e.s.d.'s in cell parameters are only used when they are defined by crystal symmetry. An approximate (isotropic) treatment of cell e.s.d.'s is used for estimating e.s.d.'s involving l.s. planes.
Refinement. Refinement of *F*^2^ against ALL reflections. The weighted *R*-factor *wR* and goodness of fit *S* are based on *F*^2^, conventional *R*-factors *R* are based on *F*, with *F* set to zero for negative *F*^2^. The threshold expression of *F*^2^ > σ(*F*^2^) is used only for calculating *R*-factors(gt) *etc*. and is not relevant to the choice of reflections for refinement. *R*-factors based on *F*^2^ are statistically about twice as large as those based on *F*, and *R*- factors based on ALL data will be even larger.The following ALERTS were generated. Each ALERT has the format test-name_ALERT_alert-type_alert-level.PLAT029_ALERT_3_C _diffrn_measured_fraction_theta_full Low ······. 0.962Noted PLAT220_ALERT_2_C Large Non-Solvent C *U*_eq_(max)/*U*_eq_(min) ··· 3.1 RatioNo atoms missing from model. Structure complete. *R* factor = 5%. PLAT241_ALERT_2_C Check High *U*_eq_ as Compared to Neighbors for C3 PLAT242_ALERT_2_C Check Low *U*_eq_ as Compared to Neighbors for Fe1Noted. PLAT341_ALERT_3_C Low Bond Precision on C—C Bonds ··············· 0.0081 A ng PLAT911_ALERT_3_C Missing # FCF Refl Between THmin & STh/*L*= 0.595 105

### Fractional atomic coordinates and isotropic or equivalent isotropic displacement parameters (Å^2^)

**Table:**

	*x*	*y*	*z*	*U*_iso_*/*U*_eq_	
C1	0.7532 (9)	0.1921 (2)	0.2707 (7)	0.0432 (16)	
H1	0.7278	0.1702	0.1871	0.052*	
C2	0.6179 (11)	0.2232 (3)	0.3360 (8)	0.063 (2)	
H2	0.4823	0.226	0.3052	0.075*	
C3	0.7118 (13)	0.2501 (2)	0.4554 (8)	0.071 (2)	
H3	0.6523	0.274	0.5199	0.086*	
C4	0.9119 (12)	0.2348 (3)	0.4607 (7)	0.062 (2)	
H4	1.0128	0.2471	0.5285	0.074*	
C5	0.9355 (9)	0.1985 (2)	0.3497 (7)	0.0461 (17)	
H5	1.0544	0.1811	0.3306	0.055*	
C6	0.4953 (8)	0.13772 (19)	0.5817 (7)	0.0342 (15)	
H6	0.3697	0.135	0.5304	0.041*	
C7	0.5441 (9)	0.1706 (2)	0.7033 (7)	0.0426 (16)	
H7	0.4561	0.1933	0.7487	0.051*	
C8	0.7447 (10)	0.16392 (19)	0.7455 (7)	0.0430 (16)	
H8	0.8162	0.1815	0.8234	0.052*	
C9	0.8213 (8)	0.1265 (2)	0.6515 (7)	0.0355 (15)	
H9	0.9537	0.1147	0.6556	0.043*	
C10	0.6672 (7)	0.10918 (18)	0.5490 (6)	0.0276 (14)	
C11	0.6982 (7)	0.07124 (18)	0.4387 (6)	0.0244 (13)	
H11	0.8338	0.0636	0.4199	0.029*	
C12	0.5657 (7)	0.04438 (18)	0.3566 (6)	0.0235 (13)	
C13	0.6178 (7)	0.00500 (18)	0.2482 (6)	0.0233 (13)	
C14	0.4696 (8)	−0.02564 (19)	0.1894 (6)	0.0285 (14)	
H14	0.3345	−0.0199	0.2162	0.034*	
C15	0.5161 (8)	−0.0640 (2)	0.0932 (6)	0.0322 (15)	
H15	0.4146	−0.0841	0.0519	0.039*	
C16	0.7119 (8)	−0.07241 (18)	0.0592 (6)	0.0292 (13)	
C17	0.8631 (8)	−0.0431 (2)	0.1144 (7)	0.0391 (16)	
H17	0.9981	−0.0497	0.0889	0.047*	
C18	0.8141 (7)	−0.0042 (2)	0.2070 (6)	0.0312 (14)	
H18	0.9162	0.0166	0.2432	0.037*	
C19	0.3559 (8)	0.05321 (19)	0.3775 (7)	0.0304 (14)	
N1	0.1881 (7)	0.05873 (18)	0.3927 (6)	0.0506 (15)	
N2	0.7683 (9)	−0.11443 (17)	−0.0399 (6)	0.0459 (14)	
O1	0.9458 (7)	−0.12327 (16)	−0.0540 (6)	0.0688 (15)	
O2	0.6336 (7)	−0.13769 (15)	−0.0995 (5)	0.0585 (13)	
Fe1	0.72165 (11)	0.17982 (3)	0.50931 (10)	0.0325 (3)	

### Atomic displacement parameters (Å^2^)

**Table:**

	*U*^11^	*U*^22^	*U*^33^	*U*^12^	*U*^13^	*U*^23^
C1	0.061 (4)	0.035 (4)	0.034 (3)	0.000 (4)	0.006 (3)	−0.003 (3)
C2	0.081 (5)	0.059 (5)	0.048 (5)	0.016 (5)	0.008 (4)	0.023 (4)
C3	0.133 (8)	0.034 (4)	0.047 (5)	0.009 (5)	0.038 (6)	0.007 (4)
C4	0.096 (6)	0.061 (5)	0.029 (4)	−0.050 (5)	0.004 (4)	−0.002 (4)
C5	0.050 (4)	0.052 (4)	0.036 (4)	−0.015 (3)	0.007 (3)	0.002 (3)
C6	0.033 (3)	0.030 (4)	0.040 (4)	−0.005 (3)	0.008 (3)	−0.003 (3)
C7	0.057 (4)	0.031 (4)	0.040 (4)	−0.001 (3)	0.022 (3)	−0.005 (3)
C8	0.065 (4)	0.026 (4)	0.038 (4)	−0.014 (3)	−0.004 (4)	−0.005 (3)
C9	0.030 (3)	0.032 (4)	0.044 (4)	−0.005 (3)	−0.007 (3)	0.002 (3)
C10	0.032 (3)	0.024 (3)	0.027 (3)	0.000 (3)	0.006 (3)	−0.005 (3)
C11	0.015 (3)	0.025 (3)	0.032 (3)	0.001 (2)	0.002 (2)	0.002 (3)
C12	0.015 (3)	0.024 (3)	0.032 (3)	−0.004 (2)	0.001 (3)	−0.003 (3)
C13	0.020 (3)	0.023 (3)	0.026 (3)	−0.002 (3)	−0.002 (2)	0.003 (3)
C14	0.022 (3)	0.030 (4)	0.033 (3)	−0.002 (3)	0.002 (3)	−0.002 (3)
C15	0.032 (4)	0.033 (4)	0.032 (3)	−0.008 (3)	−0.006 (3)	0.001 (3)
C16	0.041 (4)	0.021 (3)	0.026 (3)	0.003 (3)	0.001 (3)	0.000 (2)
C17	0.033 (3)	0.049 (4)	0.035 (4)	0.005 (3)	−0.005 (3)	−0.007 (3)
C18	0.020 (3)	0.035 (4)	0.039 (4)	−0.005 (3)	0.003 (3)	−0.015 (3)
C19	0.025 (3)	0.031 (4)	0.035 (3)	−0.004 (3)	0.001 (3)	−0.007 (3)
N1	0.024 (3)	0.055 (4)	0.073 (4)	0.001 (3)	0.001 (3)	−0.025 (3)
N2	0.061 (4)	0.037 (3)	0.040 (4)	0.012 (3)	−0.007 (3)	−0.004 (3)
O1	0.061 (3)	0.072 (4)	0.073 (4)	0.026 (3)	−0.013 (3)	−0.029 (3)
O2	0.075 (3)	0.041 (3)	0.060 (3)	−0.004 (3)	−0.008 (3)	−0.018 (2)
Fe1	0.0451 (5)	0.0244 (4)	0.0280 (4)	−0.0026 (5)	0.0043 (4)	−0.0007 (4)

### Geometric parameters (Å, °)

**Table:**

C1—C2	1.375 (8)	C8—H8	0.95
C1—C5	1.405 (8)	C9—C10	1.431 (7)
C1—Fe1	2.049 (6)	C9—Fe1	2.028 (5)
C1—H1	0.95	C9—H9	0.95
C2—C3	1.405 (9)	C10—C11	1.427 (7)
C2—Fe1	2.022 (6)	C10—Fe1	2.041 (5)
C2—H2	0.95	C11—C12	1.355 (6)
C3—C4	1.412 (9)	C11—H11	0.95
C3—Fe1	2.024 (7)	C12—C19	1.442 (7)
C3—H3	0.95	C12—C13	1.475 (7)
C4—C5	1.390 (8)	C13—C18	1.388 (7)
C4—Fe1	2.044 (6)	C13—C14	1.405 (6)
C4—H4	0.95	C14—C15	1.383 (7)
C5—Fe1	2.037 (6)	C14—H14	0.95
C5—H5	0.95	C15—C16	1.367 (7)
C6—C7	1.414 (7)	C15—H15	0.95
C6—C10	1.433 (7)	C16—C17	1.386 (7)
C6—Fe1	2.020 (5)	C16—N2	1.493 (7)
C6—H6	0.95	C17—C18	1.381 (7)
C7—C8	1.406 (8)	C17—H17	0.95
C7—Fe1	2.040 (6)	C18—H18	0.95
C7—H7	0.95	C19—N1	1.145 (6)
C8—C9	1.412 (7)	N2—O2	1.222 (6)
C8—Fe1	2.043 (6)	N2—O1	1.224 (6)
			
C2—C1—C5	107.8 (6)	C18—C13—C12	121.4 (4)
C2—C1—Fe1	69.2 (4)	C14—C13—C12	120.5 (4)
C5—C1—Fe1	69.5 (3)	C15—C14—C13	121.4 (5)
C2—C1—H1	126.1	C15—C14—H14	119.3
C5—C1—H1	126.1	C13—C14—H14	119.3
Fe1—C1—H1	126.8	C16—C15—C14	118.4 (5)
C1—C2—C3	109.3 (7)	C16—C15—H15	120.8
C1—C2—Fe1	71.3 (4)	C14—C15—H15	120.8
C3—C2—Fe1	69.7 (4)	C15—C16—C17	122.2 (5)
C1—C2—H2	125.4	C15—C16—N2	119.9 (5)
C3—C2—H2	125.4	C17—C16—N2	118.0 (5)
Fe1—C2—H2	125.2	C18—C17—C16	118.8 (5)
C2—C3—C4	106.6 (7)	C18—C17—H17	120.6
C2—C3—Fe1	69.6 (4)	C16—C17—H17	120.6
C4—C3—Fe1	70.4 (4)	C17—C18—C13	121.0 (5)
C2—C3—H3	126.7	C17—C18—H18	119.5
C4—C3—H3	126.7	C13—C18—H18	119.5
Fe1—C3—H3	124.8	N1—C19—C12	177.8 (6)
C5—C4—C3	108.2 (6)	O2—N2—O1	125.0 (5)
C5—C4—Fe1	69.9 (4)	O2—N2—C16	117.5 (5)
C3—C4—Fe1	68.9 (4)	O1—N2—C16	117.5 (5)
C5—C4—H4	125.9	C6—Fe1—C2	108.1 (3)
C3—C4—H4	125.9	C6—Fe1—C3	127.8 (3)
Fe1—C4—H4	126.9	C2—Fe1—C3	40.7 (3)
C4—C5—C1	108.1 (6)	C6—Fe1—C9	68.8 (2)
C4—C5—Fe1	70.3 (4)	C2—Fe1—C9	169.1 (3)
C1—C5—Fe1	70.3 (3)	C3—Fe1—C9	149.4 (3)
C4—C5—H5	125.9	C6—Fe1—C5	151.8 (2)
C1—C5—H5	125.9	C2—Fe1—C5	67.2 (3)
Fe1—C5—H5	125	C3—Fe1—C5	67.9 (3)
C7—C6—C10	108.4 (5)	C9—Fe1—C5	110.3 (2)
C7—C6—Fe1	70.4 (3)	C6—Fe1—C7	40.8 (2)
C10—C6—Fe1	70.1 (3)	C2—Fe1—C7	116.9 (3)
C7—C6—H6	125.8	C3—Fe1—C7	106.5 (3)
C10—C6—H6	125.8	C9—Fe1—C7	68.1 (2)
Fe1—C6—H6	125.3	C5—Fe1—C7	167.0 (2)
C8—C7—C6	108.5 (5)	C6—Fe1—C10	41.30 (19)
C8—C7—Fe1	70.0 (3)	C2—Fe1—C10	129.8 (3)
C6—C7—Fe1	68.9 (3)	C3—Fe1—C10	167.4 (3)
C8—C7—H7	125.7	C9—Fe1—C10	41.16 (19)
C6—C7—H7	125.7	C5—Fe1—C10	119.0 (2)
Fe1—C7—H7	127	C7—Fe1—C10	68.9 (2)
C7—C8—C9	107.9 (5)	C6—Fe1—C8	68.6 (2)
C7—C8—Fe1	69.8 (3)	C2—Fe1—C8	149.2 (3)
C9—C8—Fe1	69.1 (3)	C3—Fe1—C8	115.7 (3)
C7—C8—H8	126.1	C9—Fe1—C8	40.6 (2)
C9—C8—H8	126.1	C5—Fe1—C8	130.2 (3)
Fe1—C8—H8	126.6	C7—Fe1—C8	40.3 (2)
C8—C9—C10	109.0 (5)	C10—Fe1—C8	69.1 (2)
C8—C9—Fe1	70.3 (3)	C6—Fe1—C4	166.5 (3)
C10—C9—Fe1	69.9 (3)	C2—Fe1—C4	67.5 (3)
C8—C9—H9	125.5	C3—Fe1—C4	40.6 (3)
C10—C9—H9	125.5	C9—Fe1—C4	117.9 (3)
Fe1—C9—H9	125.9	C5—Fe1—C4	39.8 (2)
C11—C10—C9	122.6 (5)	C7—Fe1—C4	128.5 (3)
C11—C10—C6	131.3 (5)	C10—Fe1—C4	151.2 (3)
C9—C10—C6	106.1 (4)	C8—Fe1—C4	108.2 (2)
C11—C10—Fe1	126.2 (4)	C6—Fe1—C1	118.2 (2)
C9—C10—Fe1	68.9 (3)	C2—Fe1—C1	39.5 (2)
C6—C10—Fe1	68.6 (3)	C3—Fe1—C1	67.7 (3)
C12—C11—C10	130.5 (5)	C9—Fe1—C1	131.9 (2)
C12—C11—H11	114.7	C5—Fe1—C1	40.2 (2)
C10—C11—H11	114.7	C7—Fe1—C1	150.2 (3)
C11—C12—C19	118.9 (5)	C10—Fe1—C1	110.0 (2)
C11—C12—C13	125.1 (4)	C8—Fe1—C1	169.3 (2)
C19—C12—C13	115.9 (4)	C4—Fe1—C1	67.1 (2)
C18—C13—C14	118.1 (5)		
			
C5—C1—C2—C3	0.7 (7)	C10—C9—Fe1—C5	−111.2 (3)
Fe1—C1—C2—C3	59.6 (5)	C8—C9—Fe1—C7	−37.4 (3)
C5—C1—C2—Fe1	−58.9 (4)	C10—C9—Fe1—C7	82.6 (3)
C1—C2—C3—C4	0.5 (8)	C8—C9—Fe1—C10	−120.0 (5)
Fe1—C2—C3—C4	61.1 (5)	C10—C9—Fe1—C8	120.0 (5)
C1—C2—C3—Fe1	−60.6 (5)	C8—C9—Fe1—C4	85.8 (4)
C2—C3—C4—C5	−1.6 (7)	C10—C9—Fe1—C4	−154.3 (3)
Fe1—C3—C4—C5	59.0 (4)	C8—C9—Fe1—C1	169.1 (3)
C2—C3—C4—Fe1	−60.6 (5)	C10—C9—Fe1—C1	−70.9 (4)
C3—C4—C5—C1	2.0 (7)	C4—C5—Fe1—C6	167.6 (5)
Fe1—C4—C5—C1	60.4 (4)	C1—C5—Fe1—C6	48.9 (7)
C3—C4—C5—Fe1	−58.4 (5)	C4—C5—Fe1—C2	81.7 (5)
C2—C1—C5—C4	−1.7 (7)	C1—C5—Fe1—C2	−36.9 (4)
Fe1—C1—C5—C4	−60.4 (4)	C4—C5—Fe1—C3	37.6 (4)
C2—C1—C5—Fe1	58.8 (4)	C1—C5—Fe1—C3	−81.0 (4)
C10—C6—C7—C8	1.2 (6)	C4—C5—Fe1—C9	−109.7 (5)
Fe1—C6—C7—C8	−58.9 (4)	C1—C5—Fe1—C9	131.7 (4)
C10—C6—C7—Fe1	60.1 (4)	C4—C5—Fe1—C7	−29.4 (14)
C6—C7—C8—C9	−0.7 (7)	C1—C5—Fe1—C7	−148.0 (10)
Fe1—C7—C8—C9	−58.8 (4)	C4—C5—Fe1—C10	−154.2 (4)
C6—C7—C8—Fe1	58.2 (4)	C1—C5—Fe1—C10	87.2 (4)
C7—C8—C9—C10	−0.1 (6)	C4—C5—Fe1—C8	−68.1 (5)
Fe1—C8—C9—C10	−59.4 (4)	C1—C5—Fe1—C8	173.3 (4)
C7—C8—C9—Fe1	59.2 (4)	C1—C5—Fe1—C4	−118.6 (6)
C8—C9—C10—C11	179.9 (5)	C4—C5—Fe1—C1	118.6 (6)
Fe1—C9—C10—C11	120.3 (5)	C8—C7—Fe1—C6	120.3 (5)
C8—C9—C10—C6	0.9 (6)	C8—C7—Fe1—C2	−152.9 (4)
Fe1—C9—C10—C6	−58.7 (4)	C6—C7—Fe1—C2	86.8 (4)
C8—C9—C10—Fe1	59.6 (4)	C8—C7—Fe1—C3	−110.4 (4)
C7—C6—C10—C11	179.8 (5)	C6—C7—Fe1—C3	129.3 (4)
Fe1—C6—C10—C11	−119.9 (6)	C8—C7—Fe1—C9	37.7 (3)
C7—C6—C10—C9	−1.3 (6)	C6—C7—Fe1—C9	−82.6 (4)
Fe1—C6—C10—C9	58.9 (4)	C8—C7—Fe1—C5	−47.6 (13)
C7—C6—C10—Fe1	−60.2 (4)	C6—C7—Fe1—C5	−167.8 (10)
C9—C10—C11—C12	165.5 (5)	C8—C7—Fe1—C10	82.1 (4)
C6—C10—C11—C12	−15.8 (10)	C6—C7—Fe1—C10	−38.2 (3)
Fe1—C10—C11—C12	−108.0 (6)	C6—C7—Fe1—C8	−120.3 (5)
C10—C11—C12—C19	−0.2 (9)	C8—C7—Fe1—C4	−71.3 (5)
C10—C11—C12—C13	−178.3 (5)	C6—C7—Fe1—C4	168.5 (4)
C11—C12—C13—C18	−7.7 (8)	C8—C7—Fe1—C1	175.8 (4)
C19—C12—C13—C18	174.0 (5)	C6—C7—Fe1—C1	55.6 (6)
C11—C12—C13—C14	169.4 (5)	C11—C10—Fe1—C6	126.2 (6)
C19—C12—C13—C14	−8.8 (7)	C9—C10—Fe1—C6	−118.1 (4)
C18—C13—C14—C15	0.1 (8)	C11—C10—Fe1—C2	55.9 (6)
C12—C13—C14—C15	−177.1 (5)	C9—C10—Fe1—C2	171.5 (4)
C13—C14—C15—C16	1.6 (8)	C6—C10—Fe1—C2	−70.4 (4)
C14—C15—C16—C17	−1.6 (8)	C11—C10—Fe1—C3	92.9 (12)
C14—C15—C16—N2	178.0 (5)	C9—C10—Fe1—C3	−151.5 (11)
C15—C16—C17—C18	0.1 (8)	C6—C10—Fe1—C3	−33.3 (13)
N2—C16—C17—C18	−179.6 (5)	C11—C10—Fe1—C9	−115.7 (6)
C16—C17—C18—C13	1.6 (8)	C6—C10—Fe1—C9	118.1 (4)
C14—C13—C18—C17	−1.7 (8)	C11—C10—Fe1—C5	−27.5 (5)
C12—C13—C18—C17	175.5 (5)	C9—C10—Fe1—C5	88.2 (4)
C11—C12—C19—N1	−154 (17)	C6—C10—Fe1—C5	−153.7 (3)
C13—C12—C19—N1	24 (17)	C11—C10—Fe1—C7	163.9 (5)
C15—C16—N2—O2	6.5 (7)	C9—C10—Fe1—C7	−80.4 (3)
C17—C16—N2—O2	−173.8 (5)	C6—C10—Fe1—C7	37.7 (3)
C15—C16—N2—O1	−172.8 (5)	C11—C10—Fe1—C8	−152.8 (5)
C17—C16—N2—O1	6.9 (7)	C9—C10—Fe1—C8	−37.1 (3)
C7—C6—Fe1—C2	−110.5 (4)	C6—C10—Fe1—C8	81.0 (3)
C10—C6—Fe1—C2	130.4 (4)	C11—C10—Fe1—C4	−62.8 (7)
C7—C6—Fe1—C3	−69.7 (5)	C9—C10—Fe1—C4	52.8 (6)
C10—C6—Fe1—C3	171.2 (4)	C6—C10—Fe1—C4	170.9 (5)
C7—C6—Fe1—C9	80.6 (4)	C11—C10—Fe1—C1	15.8 (5)
C10—C6—Fe1—C9	−38.5 (3)	C9—C10—Fe1—C1	131.5 (3)
C7—C6—Fe1—C5	174.2 (5)	C6—C10—Fe1—C1	−110.4 (3)
C10—C6—Fe1—C5	55.2 (6)	C7—C8—Fe1—C6	−37.3 (3)
C10—C6—Fe1—C7	−119.1 (5)	C9—C8—Fe1—C6	82.1 (3)
C7—C6—Fe1—C10	119.1 (5)	C7—C8—Fe1—C2	52.3 (7)
C7—C6—Fe1—C8	36.9 (3)	C9—C8—Fe1—C2	171.7 (5)
C10—C6—Fe1—C8	−82.2 (3)	C7—C8—Fe1—C3	85.5 (4)
C7—C6—Fe1—C4	−42.0 (12)	C9—C8—Fe1—C3	−155.2 (4)
C10—C6—Fe1—C4	−161.0 (10)	C7—C8—Fe1—C9	−119.4 (5)
C7—C6—Fe1—C1	−152.2 (4)	C7—C8—Fe1—C5	167.4 (4)
C10—C6—Fe1—C1	88.7 (4)	C9—C8—Fe1—C5	−73.2 (4)
C1—C2—Fe1—C6	−112.7 (4)	C9—C8—Fe1—C7	119.4 (5)
C3—C2—Fe1—C6	127.5 (4)	C7—C8—Fe1—C10	−81.7 (4)
C1—C2—Fe1—C3	119.8 (6)	C9—C8—Fe1—C10	37.6 (3)
C1—C2—Fe1—C9	−41.0 (16)	C7—C8—Fe1—C4	128.7 (4)
C3—C2—Fe1—C9	−160.7 (13)	C9—C8—Fe1—C4	−111.9 (4)
C1—C2—Fe1—C5	37.6 (4)	C7—C8—Fe1—C1	−168.7 (12)
C3—C2—Fe1—C5	−82.2 (4)	C9—C8—Fe1—C1	−49.4 (14)
C1—C2—Fe1—C7	−156.0 (4)	C5—C4—Fe1—C6	−154.2 (9)
C3—C2—Fe1—C7	84.2 (5)	C3—C4—Fe1—C6	−34.4 (13)
C1—C2—Fe1—C10	−71.9 (5)	C5—C4—Fe1—C2	−80.9 (4)
C3—C2—Fe1—C10	168.3 (4)	C3—C4—Fe1—C2	38.9 (4)
C1—C2—Fe1—C8	168.9 (4)	C5—C4—Fe1—C3	−119.8 (6)
C3—C2—Fe1—C8	49.2 (7)	C5—C4—Fe1—C9	88.7 (4)
C1—C2—Fe1—C4	80.9 (4)	C3—C4—Fe1—C9	−151.5 (4)
C3—C2—Fe1—C4	−38.9 (4)	C3—C4—Fe1—C5	119.8 (6)
C3—C2—Fe1—C1	−119.8 (6)	C5—C4—Fe1—C7	171.9 (4)
C2—C3—Fe1—C6	−72.6 (5)	C3—C4—Fe1—C7	−68.3 (5)
C4—C3—Fe1—C6	170.4 (4)	C5—C4—Fe1—C10	52.3 (7)
C4—C3—Fe1—C2	−117.0 (6)	C3—C4—Fe1—C10	172.1 (5)
C2—C3—Fe1—C9	173.0 (5)	C5—C4—Fe1—C8	131.7 (4)
C4—C3—Fe1—C9	55.9 (7)	C3—C4—Fe1—C8	−108.4 (4)
C2—C3—Fe1—C5	80.2 (4)	C5—C4—Fe1—C1	−38.0 (4)
C4—C3—Fe1—C5	−36.8 (4)	C3—C4—Fe1—C1	81.8 (4)
C2—C3—Fe1—C7	−112.3 (5)	C2—C1—Fe1—C6	84.4 (5)
C4—C3—Fe1—C7	130.7 (4)	C5—C1—Fe1—C6	−156.2 (4)
C2—C3—Fe1—C10	−45.3 (13)	C5—C1—Fe1—C2	119.5 (6)
C4—C3—Fe1—C10	−162.3 (10)	C2—C1—Fe1—C3	−37.7 (4)
C2—C3—Fe1—C8	−154.5 (4)	C5—C1—Fe1—C3	81.8 (4)
C4—C3—Fe1—C8	88.4 (4)	C2—C1—Fe1—C9	170.4 (4)
C2—C3—Fe1—C4	117.0 (6)	C5—C1—Fe1—C9	−70.1 (5)
C2—C3—Fe1—C1	36.6 (4)	C2—C1—Fe1—C5	−119.5 (6)
C4—C3—Fe1—C1	−80.4 (4)	C2—C1—Fe1—C7	46.7 (7)
C8—C9—Fe1—C6	−81.4 (4)	C5—C1—Fe1—C7	166.2 (5)
C10—C9—Fe1—C6	38.6 (3)	C2—C1—Fe1—C10	129.0 (4)
C8—C9—Fe1—C2	−156.9 (13)	C5—C1—Fe1—C10	−111.6 (4)
C10—C9—Fe1—C2	−36.9 (15)	C2—C1—Fe1—C8	−148.0 (12)
C8—C9—Fe1—C3	48.1 (6)	C5—C1—Fe1—C8	−28.6 (15)
C10—C9—Fe1—C3	168.1 (5)	C2—C1—Fe1—C4	−81.9 (5)
C8—C9—Fe1—C5	128.8 (4)	C5—C1—Fe1—C4	37.6 (4)
